# Consensus statement of the European guidelines on clinical management of HIV-1 tropism testing

**DOI:** 10.1186/1758-2652-13-S4-O7

**Published:** 2010-11-08

**Authors:** LPR Vandekerckhove, AMJ Wensing, R Kaiser, F Brun-Vezinet, B Clotet, A De Luca, S Dressler, F Garcia, AM Geretti, T Klimkait, K Korn, B Masquelier, CF Perno, J Schapiro, V Soriano, A Sönnerborg, ÀM Vandamme, C Verhofstede, H Walter, M Zazzi, CA Boucher

**Affiliations:** 1 Ghent University Hospital, Infectious Diseases Unit, Ghent, Belgium; 2 University Medical Center Utrecht, Dept of Virology, Medical Microbiology, Utrecht, Netherlands; 3 University of Cologne, Institute of Virology, Cologne, Germany; 4 Bichat-Claude Bernard University Hospital, Laboratoire de Virologie, Paris, France; 5 IRSICAIXA Foundation, Retrovirology Laboratory, Badalona, Spain; 6 Catholic University Rome, Istituto di Clinica delle Malattie Infettive, Rome, Italy; 7 EATG, Berlin, Germany; 8 Hospital Universitario San Cecilio, Granada, Spain; 9 Royal Free Hampstead NHS Trust and UCL Medical School, Dept of Virology, London, UK; 10 University of Basel, Dept of Biomedicine, Inst of Medical Microbiology, Basel, Switzerland; 11 University of Erlangen, Institute of Clinical and Molecular Virology, Erlangen, Germany; 12 CHU de Bordeaux, Laboratoire de Virologie, Bordeaux, France; 13 University of Rome Tor Vergata, Dept of Experimental Medicine, Rome, Italy; 14 Sheba Medical Center, National Hemophilia Center, Tel Aviv, Israel; 15 Hospital Carlos III, Department of Infectious Diseases, Madrid, Spain; 16 Karolinska University Hospital, Div of Infectious Diseases and Clinical Virology, Stockholm, Sweden; 17 Rega Institute and University Hospitals, Lab. for Clinical and Epidemiological Virology, Leuven, Belgium; 18 University Hospital Ghent, AIDS reference laboratory, Ghent, Belgium; 19 University of Siena, Department of Molecular Biology, Siena, Italy; 20 Erasmus Medical Center, Dept of Virology, Rotterdam, Netherlands

## Introduction

Testing for HIV tropism is recommended before prescribing a chemokine receptor blocker. To date, in most European countries HIV tropism is determined using a phenotypic test. Recently, new data have emerged supporting the use of a genotypic HIV V3-loop sequence analysis as the basis for tropism determination. The European guidelines group on clinical management of HIV-1 tropism testing was established to make recommendations to clinicians and virologists.

## Methods

We searched online databases for articles from Jan 2006 until March 2010 with the terms: tropism or CCR5-antagonist or CCR5 antagonist or maraviroc or vicriviroc. Additional articles and/or conference abstracts were identified by hand searching. This strategy identified 712 potential articles and 1240 abstracts. All were reviewed and finally 57 papers and 42 abstracts were included and used by the panel to reach a consensus statement.

## Results

The panel recommends HIV-tropism testing for the following indications: i) drug-naïve patients in whom toxicity or limited therapeutic options are foreseen; ii) patients experiencing therapy failure whenever a treatment change is considered. Both the phenotypic Enhanced Trofile assay (ESTA) and genotypic population sequencing of the V3-loop are recommended for use in clinical practice. Although the panel does not recommend one methodology over another it is anticipated that genotypic testing will be used more frequently because of its greater accessibility, lower cost and shorter turnaround time. The panel also provides guidance on technical aspects and interpretation issues. If using genotypic methods, triplicate PCR amplification and sequencing testing is advised using the G2P interpretation tool (clonal model) with an FPR of 10%. If the viral load is below the level of reliable amplification, proviral DNA can be used, and the panel recommends performing triplicate testing and use of an FPR of 10%. If genotypic DNA testing is not performed in triplicate the FPR should be increased to 20%.

## Conclusions

The European guidelines on clinical management of HIV-1 tropism testing provide an overview of current literature, evidence-based recommendations for the clinical use of tropism testing and expert guidance on unresolved issues and current developments. Current data support both the use of genotypic population sequencing and ESTA for co-receptor tropism determination. For practical reasons genotypic population sequencing is the preferred method in Europe.

